# Survey of *Ixodes pacificus* Ticks in California Reveals a Diversity of Microorganisms and a Novel and Widespread *Anaplasmataceae* Species

**DOI:** 10.1371/journal.pone.0135828

**Published:** 2015-09-16

**Authors:** Mark W. Eshoo, Heather E. Carolan, Christian Massire, Danny M. Chou, Chris D. Crowder, Megan A. Rounds, Curtis A. Phillipson, Steven E. Schutzer, David J. Ecker

**Affiliations:** 1 Ibis Biosciences, an Abbott Company, Carlsbad CA, United States of America; 2 Rutgers New Jersey Medical School, Dept. of Medicine, Newark, New Jersey, United States of America; Kansas State University, UNITED STATES

## Abstract

*Ixodes pacificus* ticks can harbor a wide range of human and animal pathogens. To survey the prevalence of tick-borne known and putative pathogens, we tested 982 individual adult and nymphal *I*. *pacificus* ticks collected throughout California between 2007 and 2009 using a broad-range PCR and electrospray ionization mass spectrometry (PCR/ESI-MS) assay designed to detect a wide range of tick-borne microorganisms. Overall, 1.4% of the ticks were found to be infected with *Borrelia burgdorferi*, 2.0% were infected with *Borrelia miyamotoi* and 0.3% were infected with *Anaplasma phagocytophilum*. In addition, 3.0% were infected with *Babesia odocoilei*. About 1.2% of the ticks were co-infected with more than one pathogen or putative pathogen. In addition, we identified a novel *Anaplasmataceae* species that we characterized by sequencing of its 16S rRNA, *groEL*, *gltA*, and *rpoB* genes. Sequence analysis indicated that this organism is phylogenetically distinct from known *Anaplasma* species with its closest genetic near neighbors coming from Asia. The prevalence of this novel *Anaplasmataceae* species was as high as 21% at one site, and it was detected in 4.9% of ticks tested statewide. Based upon this genetic characterization we propose that this organism be called ‘*Candidatus* Cryptoplasma californiense’. Knowledge of this novel microbe will provide awareness for the community about the breadth of the *I*. *pacificus* microbiome, the concept that this bacterium could be more widely spread; and an opportunity to explore whether this bacterium also contributes to human or animal disease burden.

## Introduction

In the western United States *Ixodes pacificus* ticks are the vector of *Borrelia burgdorferi*, the causative agent of Lyme disease [1,2]. *I*. *pacificus* ticks are also vectors for several other vector-borne pathogens including *Borrelia miyamotoi* and *Anaplasma phagocytophilum*. In addition, *I*. *pacificus* ticks have been shown to carry *Spiroplasma ixodetis* [3], a microorganism that has no known role in human disease. Broad-range PCR and electrospray ionization mass spectrometry (PCR/ESI-MS) can detect multiple pathogens in a single test including tick-borne pathogens [4–14]. PCR/ESI-MS can also detect novel and uncharacterized organisms [10,12,15–19]. We have previously used PCR/ESI-MS to characterize a large collection of ticks, including *I*. *pacificus* ticks from California, for the prevalence of *B*. *miyamotoi* [6]. Most studies of *I*. *pacificus* ticks have employed tests designed to detect single pathogens and not co-infections. In this study we used PCR/ESI-MS to characterize the breadth of microorganisms carried by *I*. *pacificus* collected from throughout the state of California. In the ticks analyzed, we detected several previously described endemic zoonotic pathogens as well as *Babesia odocoilei*, a protozoan not previously known to be carried by *I*. *pacificus*. Many of the microbes carried by ticks are obligate intracellular microbes and have not been cultured but have been identified and characterized genetically such as ‘*Candidatus* Rickettsia andeanae’, ‘*Candidatus* Rickettsia vini’ and ‘*Candidatus* Neoehrlichia mikurensis' [20–23]. In our study we identified and genetically characterized a novel and widespread *Anaplasmataceae* species which is genetically most closely related to isolates from Asia.

## Methods and Materials

### Broad-range PCR/ESI-MS detection of tick-borne microorganisms

Broad-range PCR employs PCR primers designed to generate an amplicon from a wide range of microbes. Depending upon the organism(s) present in the sample the resulting amplicons will be comprised of differing composition of bases (the number of A’s, G’s, C’s and T’s aka base count). When these amplicons are analyzed by electrospray ionization mass spectrometry the base count of the amplicons can be determined and compared to a database to identify the organism(s) present. Nucleic acid extracts from ticks were screened for tick-borne microorganisms with three variations of a broad-range PCR/ESI-MS assay (**Table 1**). Primer set A includes 16 primer pairs designed to detect and identify a wide and diverse range of bacterial species. Primer set B includes two primer pairs designed to detect all *Babesia* species [5]. Primer set C consists of nine primer pairs designed to detect a wide range of bacterial and protozoan tick-borne pathogens that were previously described [5]. PCR amplicons were analyzed by electrospray ionization followed by time of flight mass spectrometry system (Abbott Laboratories, Des Plaines IL) as previously described [10,13,14,24–27].

**Table 1:** PCR/ESI-MS primer and gene targets.

Primer set	Primer Pair ID	Primer ID	Primer Sequence	Target	Target Clade/Genus
C	BCT3514	BCT8235F	TTTGGTACCACAAAGGAATGGGA	*rpoC*	All Spirochaetes
		BCT8236R	TGCGAGCTCTATATGCCCCAT	* *	
C	BCT3570	BCT8336F	TGCATGCAGATCATGAACAGAATGC	*gltA*	Alphaproteobacteria
		BCT8337R	TCCACCATGAGCTGGTCCCCA	* *	
B & C	INV4443	INV10034F	TGCGCAAATTACCCAATCCTGACAC	*18S rRNA*	All *Babesia* spp.
		INV10035R	TCCAGACTTGCCCTCCAATTGGTA	* *	
B & C	INV4855	INV10812F	TGAGAGAAATCGTACACATTCAAGCGGG	*β-tubulin*	All *Babesia* spp.
		INV10813R	TCCATGTTCGTCGGAGATGACTTCCCA	* *	
A, B & C	BCT3511	BCT8229F	TGCATTTGAAAGCTTGGCATTGCC	*gyrB*	All *Borrelia* spp.
		BCT8230R	TCATTTTAGCACTTCCTCCAGCAGAATC	* *	
A, B & C	BCT3517	BCT8241F	TGCTGAAGAGCTTGGAATGCA	*flagellin*	All *Borrelia* spp.
		BCT8242R	TACAGCAATTGCTTCATCTTGATTTGC	* *	
A, B & C	BCT2328	BCT5602F	TGAGGGTTTTATGCTTAAAGTTGGTTTTATTGGTT	*asd*	*F*. *tularensis*
		BCT5603R	TGATTCGATCATACGAGACATTAAAACTGAG	* *	
A, B & C	BCT1083	BCT2764F	TAAGAGCGCACCGGTAAGTTGG	*RNaseP RNA*	All *Rickettsia* spp.
		BCT2763R	TCAAGCGATCTACCCGCATTACAA	* *	
A, B & C	BCT3575	BCT8346F	TGCATCACTTGGTTGATGATAAGATACATGC	*rpoB*	Alphaproteobacteria
		BCT8347R	TCACCAAAACGCTGACCACCAAA	* *	
A & B	BCT3515	BCT8237F	TCCACAAGGTGGTGGTGAAGG	*rplB*	All Spirochaetes
		BCT8238R	TCGGCTGTCCCCAAGGAG	* *	
A & B	BCT3519	BCT8245F	TCGAATAATGTTATTGAGTTTAGATCTTTTGGTAC	*hbb*	All *Borrelia* spp.
		BCT8246R	TGGACGAAAATACGCAACATGATGATC	* *	
A & B	BCT2332	BCT5610F	TCAGCTAGACCTTTTAGGTAAAGCTAAGCT	*galE*	*F*. *tularensis*
		BCT5625R	TCTCACCTACAGCTTTAAAGCCAGCAAAATG	* *	
A & B	BCT1084	BCT2765F	TCCACCAAGAGCAAGATCAAATAGGC	*RNaseP RNA*	All *Rickettsia* spp.
		BCT2763R	TCAAGCGATCTACCCGCATTACAA	* *	
A & B	BCT3569	BCT8334F	TGCATGCAGATCATGAACAAAATGC	*gltA*	Alphaproteobacteria
		BCT8335R	TCCATGTGCTGGTCCCCA	* *	
A & B	BCT1079	BCT2717F	TCGCCGTGGAAAAATCCTACGCT	*icd*	*Coxiella burnetii*
		BCT2718R	TAGCCTTTTCTCCGGCGTAGATCT	* *	
A & B	BCT1080	BCT2719F	TCAGTATGTATCCACCGTAGCCAGTC	*IS1111A*	*Coxiella burnetii*
		BCT2720R	TAAACGTCCGATACCAATGGTTCGCTC	* *	
A & B	BCT3565	BCT8326F	TGTATATGTTGAAACTATCTCACATGGTTGATGA	*rpoB*	All *Spiroplasma* spp.
		BCT8327R	TGTGCTTTTCCTCCAAATGGTTGTTG	* *	
A & B	BCT3568	BCT8332F	TGCTTTTATTGTTGGCCAGATGGG	*traE-like ATPase*	All *Spiroplasma* spp.
		BCT8333R	TGGCAAAACTACCATATTCGCGTTG	* *	
A & B	BCT348	BCT1393F	TTTCGATGCAACGCGAAGAACCT	*16S rRNA*	All bacteria
		BCT1370R	TACGAGCTGACGACAGCCATG	* *	
A & B	BCT360	BCT1386F	TCTGTTCTTAGTACGAGAGGACC	*23S rRNA*	All bacteria
		BCT1402R	TTTCGTGCTTAGATGCTTTCAG	* *	

### Tick collection and DNA extraction

*I*. *pacificus* ticks were collected from 2007 to 2009 in thirteen counties throughout the state of California by flagging (S1 Fig). All ticks tested were adults with the exception of 126 nymphal ticks collected in Sonoma County. All ticks were visually pre-identified as *I*. *pacificus*, and the species was confirmed in 96.9% of the ticks tested by the detection of the *Rickettsia* endosymbiont of *I*. *pacificus* [10]. No ethical clearance was required to conduct research on invertebrate ectoparasites. All samples were submitted by private collectors or collected by county vector control agencies from locations that did not require specific permission. These studies did not involve endangered or protected species. Ticks were either alive, frozen, or ETOH preserved prior to DNA extraction using a modified Qiagen column-based protocol described previously [28] with the substitution of the Qiagen DNAeasy columns (Qiagen, Valencia, CA) for the Qiagen Virus MinElute columns (Qiagen, Valencia, CA). A negative extraction control consisting of lysis buffer was included with each set of extractions and carried through the analysis to monitor for potential contamination.

### Sequence confirmation of *Anaplasmataceae* detections

Nucleic acid extracts from three ticks (collected from different California counties) that showed the novel PCR/ESI-MS microorganism signature were selected for further sequence analysis. The 16S rRNA, *groEL*, *gltA*, and *rpoB* genes were sequenced by single molecule real-time DNA sequencing using the RS II DNA sequencer (Pacific Biosciences, Menlo Park CA). In addition, a tick positive for *A*. *phagocytophilum* from Placer County, CA was also selected for sequence analysis. The previously published primer pair 27F/1492R was used to amplify a 1,459-bp region of the 16S rRNA gene [29]. The g*roEL* gene was amplified using a primer pair targeting an 851-bp region of the *groEL* gene present in *Anaplasma*, ‘*Candidatus* Neoehrlichia’, and *Ehrlichia* species (F-groEL: ATCTCTAAAGCTAAAGCTGCTGG and R-groEL: ACACCAACCTTAAGTACAGCAAC). The *gltA* gene was amplified using a primer pair derived from the published F4b-R1b primer pair [30] (F-gltA: ACCGGGTTTTATGTCTACTGC and R-gltA: CACGATGACCAAAACCCAT). The *rpoB* gene was amplified using primers designed to amplify a 600–650-bp region across *Rickettsiales* (F-rpoB: ATCGTTCCTGTTGAAGATATGCC and R-rpoB: GCAATGCCCAGCATTCCAT). PCR amplification of the target genes was performed in a 50-μL reaction with 1 unit of Platinum Taq DNA Polymerase High Fidelity, 1x High Fidelity PCR Buffer, 2 mM MgSO_4_, 0.2 mM dNTP mix, and 250 nM of each primer (Life Technologies, Carlsbad, CA). All PCR amplification reactions were performed on the MJ Dyad thermocycler (Bio-Rad Inc., Hercules, CA). The following PCR conditions were used to amplify the 16S rRNA gene locus: 94°C for 2 min, followed by 40 cycles of 94°C for 15 s, annealing at 47°C for 30 s, and 68°C for 2 min for cycle extension. The cycle ended with a final extension of 4 min at 68°C. Amplification of the *groEL and gltA* loci was performed using the same conditions with the exceptions of a 50°C annealing temperature and a 1.5-min cycle extension time. PCR amplification of the *rpoB* locus was performed as described for the *groEL* and *gltA* loci except that a 1-min extension time was used. PCR products from each sample were purified using the Qiagen MinElute PCR Purification Kit. After purification, libraries were prepared using 500 ng of amplicons and a modified version of Pacific Biosciences library preparation protocol that consisted of end-repair, adapter ligation, and exonuclease treatment of the PCR amplicons. The final sequencing library was purified using the Qiagen MinElute PCR purification kit. One SMRT cell was used to sequence each amplicon library following diffusion loading. Sequencing data were analyzed using Pacific Bioscience’s RS_Long_Amplicon_Analysis.1 protocol in the SMRT Analysis v2.2.0 software package to cluster, error correct, filter out chimeras, and build consensus sequences. Sequences were deposited in GenBank under accession numbers KP276585 to KP276611.

### Sequence alignments and phylogenetic analyses

Alignments of the full-length 16S rRNA, *gltA*, *groEL*, and *rpoB* were constructed from representative *Anaplasmataceae* sequences using data from complete genomes or type strains. Sequences GU075704 and JN715833 were identified by preliminary BLAST searches as the closest relatives of the novel *Anaplasmataceae* species and were included in the ribosomal alignment. Sequence alignments were generated using ClustalW and were manually curated using secondary structure pairing constraints with Bioedit software [31] (S1, S2, S3 and S4 Alignments) to yield, after exclusion of the primer sequences and non-conserved indels, ungapped alignments for 16S rRNA (1,347 bp, N = 26 sequences), *groEL* (804 bp, N = 23), *gltA* (720 bp, N = 23) and *rpoB* (555 bp, N = 19) (S1, S2, S3 and S4 Text). Identity score matrices were generated with Bioedit from the curated alignments (S1, S2, S3 and S4 Data). Phylogenetic analyses were performed using the online program suite at www.Phylogeny.fr using the “A la Carte” menu [32] on the curated alignments. Maximum-likelihood trees were constructed as described [33,34] and edited using TreeDyn [35].

## Results

### Survey of tick-borne pathogens by broad-range PCR/ESI-MS

A total of 982 individual ticks were tested by broad-range PCR/ESI-MS for the presence of tick-borne pathogens and other microorganisms. All ticks tested were adults except for 126 nymphal ticks collected at a single site in Sonoma County. The summary of the non-endosymbiont microorganism detections is reported in **Table 2**. The *Rickettsia* endosymbiont of *I*. *pacificus* [10] was detected in 96.9% (952/982) of the ticks tested (**Table 3**), confirming the tick species assignments. In addition to the endosymbiont, at least one tick-borne microorganism was detected in 14.9% of the ticks surveyed (146/982), with co-infections detected in 1.2% of the ticks (**Table 2**).

**Table 2:** Prevalence of microorganisms in *I*. *pacificus* ticks in California by PCR/ESI-MS.

California County	Collection Site	Primer Set	Number of ticks tested	*B*. *miyamotoi*	*B*. *burgdorferi*	*Rickettsia sp*.	*B*. *odocoilei*	*A*. *phagocytophilum*	*'Ca*. C. californiense'	*S*. *ixodetis*	*B*. *miyamotoi & B*. *odocoilei*	*B*. *burgdorferi & A*. *phagocytophilum*	*B*. *burgdorferi & Rickettsia sp*.	*B*. *odocoilei & Rickettsia sp*.	*B*. *burgdorferi & S*. *ixodetis*	*'Ca*. C. californiense' *& B*. *odocoilei*	*'Ca*. C. californiense' *& Rickettsia sp*.
Alameda	Lake Temescal regional recreation area	B	22	1	0	1	1	0	0	0	0	0	0	0	0	0	0
Del Norte	Patrick's Creek	B	15	0	0	0	0	0	1	0	0	0	0	0	0	0	0
Del Norte	Patrick's Creek	C	18	0	0	0	0	0	0	ND	0	0	0	0	ND	0	0
Glenn	Ivory Mill Road, Elk Creek (24), Unknown (20)	A	44	0	0	2	ND	0	1	0	ND	0	0	ND	0	ND	0
Humboldt	West End Rd, Arcata	A	1	0	0	0	ND	0	0	0	ND	0	0	ND	0	ND	0
Humboldt	West End Rd, Arcata	B	13	0	0	0	3	0	0	0	0	0	0	0	0	0	0
Humboldt	Lord Ellis Summit	C	60	0	0	1	0	0	0	ND	0	0	0	0	ND	0	0
Lake	9 various trails	A	129	0	1	8	ND	0	9	1	ND	0	0	ND	1	ND	0
Marin	Olompali State Park	A	52	1	0	2	ND	0	3	3	ND	0	0	ND	0	ND	0
Marin	China Camp State Park	B	33	0	0	0	0	0	2	3	0	1	0	1	0	0	0
Mendocino	Russian Gulch State Park	B	5	0	0	0	0	0	0	0	0	0	0	0	0	0	0
Mendocino	Mendicino Angelo Preserve	C	52	0	0	0	0	0	7	ND	2	0	0	0	ND	1	0
Napa	Bothe State Park	B	65	10	0	3	1	0	5	2	0	0	0	0	0	1	1
Orange	Aliso & Wood Canyons Wilderness Park	A	15	0	0	0	ND	0	1	0	ND	0	0	ND	0	ND	0
Placer	Stevens Trail Colfax	A	147	2	2	12	ND	2	2	0	ND	0	1	ND	0	ND	0
Placer	Drivers Flat, Foresthill	B	103	1	3	5	3	0	9	3	1	0	0	0	0	1	0
San Bernardino	Boneyard Flats/Hwy 38	B	18	0	0	0	1	0	0	0	0	0	0	0	0	0	0
Santa Cruz	Nisene Marks State Park	A	20	0	0	0	ND	0	0	0	ND	0	0	ND	0	ND	0
Santa Cruz	Nisene Marks State Park	B	44	0	0	0	0	0	4	1	0	0	0	0	0	0	0
Sonoma	Sonoma Development Center Camp	B	44	0	3	1	0	0	0	2	1	0	0	0	0	0	0
Sonoma	Sonoma Development Center Camp	C	82	1	2	2	0	0	0	ND	0	0	0	0	ND	0	0
**All locations**			**982**	**16**	**11**	**37**	**9**	**2**	**44**	**15**	**4**	**1**	**1**	**1**	**1**	**3**	**1**
Total infection rate (%)*			** **	**2.0%**	**1.4%**	**4.1%**	**3.0%**	**0.3%**	**4.9%**	**2.1%**							

ND = Not Determined

*includes co-infections, total number of ticks test adjusted for *S*. *ixodetis* and *B*. *odocoilei*.

**Table 3:** PCR/ESI-MS signatures of *Rickettsiaceae* and *Anaplasmataceae* species.

	Base counts from indicated primer pair ^a^							
Species detected	BCT3569	BCT3570	BCT3575	BCT348	BCT360	BCT3515	BCT1083	BCT1084
	(*gltA*)	(*gltA*)	(*rpoB*)	(*16S rRNA*)	(*23S rRNA*)	*(rplB)*	*(rnpB)*	*(rnpB)*
*Rickettsia endosymbiont of I*. *scapularis*	A30G27C32T33	A28G30C32T35	No detection	A26G31C32T31	A34G37C27T24	A17G21C16T14	A42G32C30T31	A26G21C21T23
*Rickettsia endosymbiont of I*. *pacificus*	A30G27C31T34	A28G30C31T36	No detection	A25G32C34T29	A34G37C27T24	A17G21C16T14	A42G32C30T31	A26G21C21T23
*Rickettsia sp*., *spotted fever group*	A31G26C30T35	A28G30C32T35	No detection	A25G32C33T30	A33G38C26T25	A16G21C16T15	A42G33C31T31	A25G22C21T25
*A*. *phagocytophilum*	A28G34C27T33	Not determined	A22G33C20T37	A26G32C31T30	A32G35C25T30	A20G22C13T13	No detection	No detection
*‘Ca*. C. californiense’	A28G40C23T31	No detection	A22G34C20T36	A27G30C31T31	A32G35C25T30	No detection	No detection	No detection

^a^ The gene target is given in parentheses

Though our *Babesia* primers are able to detect a wide range of *Babesia* species, the only species detected in California ticks was *B*. *odocoilei*, found in 3% (18) of the 574 ticks tested for *Babesia*. Not all of the specimens were tested due to limited nucleic acid extracts. This organism is known to be transmitted by *Ixodes scapularis* but has not previously been detected in *I*. *pacificus* ticks. *Borrelia miyamotoi* was found in 2% of the ticks tested, which we have previously reported [6]. *Borrelia burgdorferi* was found in 1.4% of the ticks, consistent with infection rates that have been reported by others [36]. In this study, *Anaplasma phagocytophilum* was detected in only 3 of the 982 ticks tested. Two of the *A*. *phagocytophilum*-positive ticks came from the same site in Placer County and the third detection was in a *B*. *burgdorferi* co-infected tick from Marin County. *Spiroplasma ixodetis* was found in 2% (16/770) of the ticks tested using the primer set that detects this organism. In addition to the *Rickettsia* endosymbiont of *I*. *pacificus*, 37 ticks tested yielded a PCR/ESI-MS base count signature similar, but not identical, to that of a spotted fever group *Rickettsia* species (**Table 3**). Though these signatures are highly consistent with one or more *Rickettsia* species, further analysis is needed to confirm the identity.

### Identification and characterization of a novel *Anaplasmataceae* species

The most prevalent non-endosymbiont was a novel bacteria found in 4.9% (48/982) of the ticks tested. Ticks from China Camp State Park in Marin County had the highest observed infection rate of 22% (7/33) for this organism. The bacterium was detected in ticks collected throughout the state with detections in ticks collected in Aliso & Wood Canyons Wilderness Park in Orange County and at Patrick Creek in Del Norte County in the far northern part of the state. The PCR/ESI-MS signature of this novel bacterium was similar to that of *A*. *phagocytophilum* (**Table 3**). Given the breadth of coverage provided by the primer pairs used in the PCR/ESI-MS assay, this signature is consistent with the detection of a novel *Anaplasmataceae* species. Three representative tick samples containing this signature (samples CP-1, MR-9, and CC-14 from Napa, Placer, and Marin Counties, respectively) were selected for DNA sequence analysis. We also selected for sequencing one of the three ticks positive for *A*. *phagocytophilum* (sample MR-23 from Placer County) for sequencing. The four loci sequenced, the 16S rRNA gene, *gltA*, *groEL and rpoB*, were chosen to clarify the relationship of the novel species with named *Anaplasma*, *Ehrlichia* and ‘*Candidatus* Neoehrlichia’ species.

**Fig 1:**
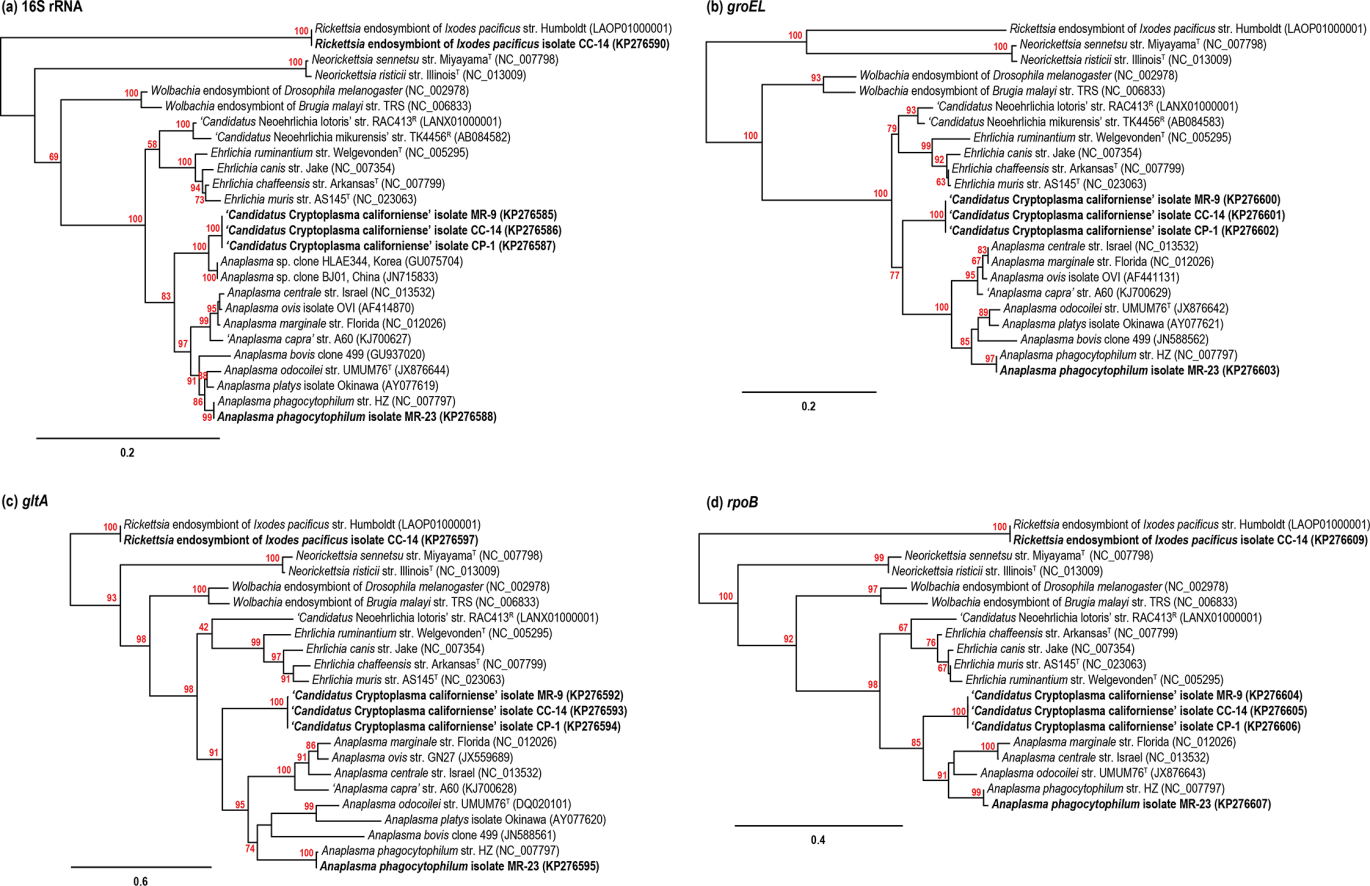
Maximum-likelihood tree relationships among know *Anaplasmataceae* species and novel microorganism identified in this study inferred from (a) 16S rRNA, (b) *groEL*, (c) *gltA*, and (d) *rpoB* sequences. Sequences determined in this study are highlighted in bold. Numbers at nodes indicate percentages of bootstrap support based on 100 replicates. The horizontal bars correspond to substitutions per nucleotide position in panel (a) and to substitutions per amino acid position in panels (b-d).

Phylogenetic trees were constructed based on the four sequenced genes and publicly available sequences of representative *Anaplasmataceae* and more distantly related Rickettsiales (Fig 1A–1D). The four single gene trees are largely congruent and show previously established features like the monophyly of the genera *Anaplasma*, *Ehrlichia*, and *‘Candidatus* Neoehrlichia’ [23,37]. The split of *Anaplasma* species into two branches is also shown in the 16S rRNA, *groEL* and *gltA* trees, where all named *Anaplasma* species are represented [38]. Minor differences are observed within the exact branching order of species within the established clades, in particular for *A*. *bovis* [39]. Each tree firmly places the novel *Anaplasmataceae* microorganism on the branch leading to the *Anaplasma* clade [37]. The four gene sequences of the *A*. *phagocytophilum* isolate (MR-23) had 99.4% to 100% identity to the *A*. *phagocytophilum* genome sequence (GenBank NC_007797). The observed deviations from the reference genome sequence in the 16S rRNA gene (1 SNP) and *gltA* (2 SNPs) were shared with independent sequences of California isolates of *A*. *phagocytophilum* (AF172167, AF304137), suggesting that these differences are mostly likely unique to isolates from California.

The sequences of the genes from the three samples of the novel *Anaplasmataceae* shared 99.5% to 100% sequence identity, confirming the detection of a common species in the three samples. The closest 16S rRNA sequence found in GenBank (98.0% identity or 1320/1347 identical positions) came from an uncultured *Anaplasma* sp. isolated from *Haemaphysalis longicornis* ticks from Jeju Island, Korea (accession numbers GU075699 to GU075704) [40] and Beijing, China (JN715833) (Fig 1). No *groEL*, *gltA*, or *rpoB* sequence is currently available for those isolates. The 16S rRNA sequence from our novel *Anaplasmataceae* bacterium shared 93.4% to 94.2% identity with characterized *Anaplasma* species and 91.1% to 91.6% identity with characterized *Ehrlichia* species (Fig 1A). In comparison, previously characterized *Anaplasma* species share over 95.2% identity across the 16S rRNA gene and only 90.4% to 92.9% identity with *Ehrlichia* species. The *groEL*, *gltA*, and *rpoB* sequences from the novel microorganism had relatively low homology with other sequences in GenBank; the *groEL* sequences were slightly more similar to *Ehrlichia* than to *Anaplasma* sequences. Accordingly, the four gene trees place the novel California isolate (together with the related Chinese and Korean clones for 16S rRNA) on an intermediate branch that predates the radiation of all validated *Anaplasma* species (Fig 1A–1D).

### Phylogenetic analysis of the *Rickettsia* endosymbiont of *I*. *pacificus*

In our next generation DNA sequencing of the four ticks with *Anaplasma* we also determined the sequences of the *Rickettsia* endosymbiont 16S rRNA (GI: KP276589, KP276590 and KP276591), *gltA* (GI: KP276596, KP276597, KP276598, and KP276599), and *rpoB* (GI: KP276608, KP276609, KP276610, and KP276611) loci from four ticks. The *groEL* locus was not sequenced as it was not amplified due to primer design. No sequence variation was detected in the four tick specimens sequenced, and the sequences of the *I*. *pacificus* endosymbiont shared 100% identity with the recently published genome sequence. (Fig 1A–1D).

## Discussion

In this study of the microorganisms carried by *I*. *pacificus* ticks, we found *B*. *burgdorferi* and *B*. *miyamotoi* at frequencies consistent with those found by others [41]. Our broad-range PCR primers are capable of detecting and distinguishing a wide range of *Borrelia* species including *B*. *americana*, *B*. *bissettii*, *and B*. *californiensis* previously detected in *I*. *pacificus* ticks [42–44]. Only *B*. *miyamotoi* and *B*. *burgdorferi* were observed in this study. As the study was performed over the course of several years, we were able to improve the throughput of the assay by reducing the number of primers used from 16 to 8 without affecting sensitivity or breadth of coverage for tick-borne pathogens.

This is the first observation of the protozoan parasite *Babesia odocoilei* in *I*. *pacificus* ticks. We detected this organism in 3.0% of the ticks tested from areas ranging from Humboldt County in the far northern part of California to San Bernardino County in Southern California, indicating that this pathogen is probably widespread among its known Cervidae and Bovidae hosts [45] in the state. *B*. *odocoilei* is known to be widely distributed in *I*. *scapularis* ticks throughout the eastern United States [45,46], where it is has been reported from as far north as Saskatchewan, Canada [47] to as far south as Tennessee [48] and is not known to cause human illness.

Approximately 4% of the ticks were infected with a microorganism with a base count signature consistent with a spotted fever group *Rickettsia* that was distinct from the characterized *Rickettsia* endosymbiont of *I*. *pacificus*. Further analysis is needed to determine whether this signature represents a novel spotted fever group *Rickettsia* or a genotypic variant of a known species.

Other than the endosymbiont, the most prevalent organism we found in the ticks was a previously undescribed *Anaplasmataceae* species; the base count signature of this novel species was detected in 4.9% of ticks analyzed. Analyses of the 16S rRNA gene, *groEL*, *gltA*, and *rpoB* sequences from three samples concur to place this novel organism in the *Anaplasma*/*Ehrlichia*/‘*Candidatus* Neoehrlichia’ clade, in a position ancestral to all known *Anaplasma* species. The 16S rRNA sequence was found to share a maximum of 94.2% identify with any named *Anaplasma* species (*Anaplasma platys* isolate Okinawa, AY077619), well below the threshold of 97% conservatively associated with distinct species. Indeed, this less than 95% homology indicates a distinct genus may also be warranted [49]. As different bacterial clades may evolve at differing rates it is important to look at the clade of interest and determine the precedence for establishing new genera. In the examination of the family *Anaplasmataceae* the now well-established ‘*Candidatus* Neoehrlichia’ taxon was recognized as a genus distinct from the related *Ehrlichia* genus following analysis of the 16S rRNA gene and *groEL* sequences [50]. With the recognition of a second ‘*Candidatus* Neoehrlichia’ species and further sequencing of the *gltA* and *rpoB* genes, the ‘*Candidatus* Neoehrlichia’ and *Ehrlichia* genera share an average sequence identity of 93.4%, 88.2%, 59.5% and 86.4% across 16S rRNA, *groEL*, *gltA* and *rpoB* (S1, S2, S3 and S4 Data). In comparison, the average identity computed between the novel California isolate and the established *Anaplasma* species is 93.6%, 84.7%, 58.6% and 83.0% across the same loci. In other words, the divergence seen between the California isolate and all named *Anaplasma* species is greater than the divergence seen between the established genera *Ehrlichia* and ‘*Candidatus* Neoehrlichia’, therefore providing strong phylogenetic evidence for the recognition of a new taxon at the genus level.

A survey of available 16S rRNA gene sequences for uncharacterized *Anaplasmataceae* led to the identification of several 16S rRNA sequences that are closely related to our novel *Anaplasmataceae* species, suggesting that this new genus may be globally widespread. For example, isolates from Korea and China with near full-length 16S sequences were found to have the closest identity to our isolate at 97.9%. Several groups have reported finding “*Anaplasma*-like” partial 16S rRNA gene sequences in ticks and animals. Though these available 16S sequences are comparatively short (~200–274 nt), they show higher levels of homology to our novel *Anaplasmataceae* than to named *Anaplasma* species. For example, GenBank sequence GU734325, obtained from an extract from an *Ixodes ricinus* tick collected in the outskirts of Paris [50], has 270/274 identity (98.6%) with the 16S sequence of our novel *Anaplasmataceae*. Moreover, this same sequence was also identified in a survey of tick populations in Tunisia and Morocco (GenBank AY672415, AY672420) [51] and in a survey of rodents from Slovakia (GenBank EF121953, EF121954) [52]. These studies hint at the dissemination in East Asia, Europe, and North Africa of at least two novel *Anaplasmataceae* species more closely related to the novel California species than to characterized *Anaplasma* species. In summary, the evidence presented indicates that the bacterium identified in the present study represents a lineage distinct from known *Anaplasma* species. In agreement with the guidelines for the provisional naming of partially characterized species [53], we propose the name ‘*Candidatus* Cryptoplasma californiense ‘which should provide a clearer basis for future species recognition and naming. Further studies are needed to culture this organism and to determine its range of hosts.

### Description of ‘*Candidatus* Cryptoplasma californiense’ Eshoo et al. 2015

Cryptoplasma (Cryp.to.plas’ma. Gr. adj. *kruptos*, hidden; Gr. neut. n. *plasma*, anything formed or molded, image, figure; N.L. neut. n. *Cryptoplasma*, a thing (a bacterium) of hidden form; ca.li.for.ni.en′se. L. neut. adj. californiense, pertaining to the State of California, where the organism was found). Members of the α-Proteobacteria, placed phylogenetically within the family *Anaplasmataceae*. Not cultivated. Parasitic to *Ixodes pacificum* ticks.

‘*Candidatus* Cryptoplasma californiense’ [(α -Proteobacteria) NC; G-; U; NAS (GenBank no. KP276586), oligonucleotide sequence complementary to unique region of 16S rRNA gene 5’- TGGCTTGCCATAAAAGAGTTTAG – 3’; P (*Ixodes pacificum* ticks); M]. Eshoo et al., this study.

## Supporting Information

S1 Alignment Raw sequence alignment of the 16S rRNA for the 26 sequences included in the phylogenetic analysis. Sequences of the PCR/ESI-MS primers and sequencing primers are reported in the top two rows. The analysis mask indicates with ‘N’ the positions included in the analysis.
(FSA)

S2 Alignment Raw sequence alignment of the *groEL* gene for the 23 sequences included in the phylogenetic analysis. Sequences of the sequencing primers are reported in the top row. The analysis mask indicates with ‘N’ the 804 nucleotide positions included in the analysis. Positions were aligned with respect of the encoded protein sequence.
(FSA)

S3 Alignment Raw sequence alignment of *gltA* for the 23 sequences included in the phylogenetic analysis. Sequences of the PCR/ESI-MS primers and sequencing primers are reported in the top rows. The analysis mask indicates with ‘N’ the 720 nucleotide positions included in the analysis. Positions were aligned with respect of the encoded protein sequence.
(FSA)

S4 Alignment Raw sequence alignment of *rpoB* for the 19 sequences included in the phylogenetic analysis. Sequences of the PCR/ESI-MS primers and sequencing primers are reported in the top rows. The analysis mask indicates with ‘N’ the 555 nucleotide positions included in the analysis. Positions were aligned with respect of the encoded protein sequence.
(FSA)

S1 Data Identity score matrix computed across the 1,347 nucleotide positions of the *16S rRNA* alignment (see S1 Text). (XLSX)

S2 Data Identity score matrix computed across the 268 amino acid positions of the *groEL* alignment (see S2 Text). (XLSX)

S3 Data Identity score matrix computed across the 240 amino acid positions of the *gltA* alignment (see S3 Text). (XLSX)

S4 Data Identity score matrix computed across the 185 amino acid positions of the *rpoB* alignment (see S4 Text). (XLSX)

S1 Fig Dispersion of the collection sites (red dots) across thirteen California counties. Dot size is proportional to the number of isolates collected.
(TIF)

S1 Text Trimmed alignment of the 1,347 nucleotide positions of the 16S rRNA for the 26 sequences included in the phylogenetic analysis. (TXT)

S2 Text Trimmed alignment of the 268 amino acid positions of *groEL* for the 23 sequences included in the phylogenetic analysis. (TXT)

S3 Text Trimmed alignment of the 240 amino acid positions of *gltA* for the 23 sequences included in the phylogenetic analysis. (TXT)

S4 Text Trimmed alignment of the 185 amino acid positions of *rpoB* for the 19 sequences included in the phylogenetic analysis. (TXT)
