# Supplementary figures and images for: Survey of Ixodes pacificus Ticks in California Reveals a Diversity of Microorganisms and a Novel and Widespread Anaplasmataceae Species
Source: PLoS One. 2015 Sep 16;10(9):e0135828. doi: 10.1371/journal.pone.0135828 (PMC4574436; doi:10.1371/journal.pone.0135828)

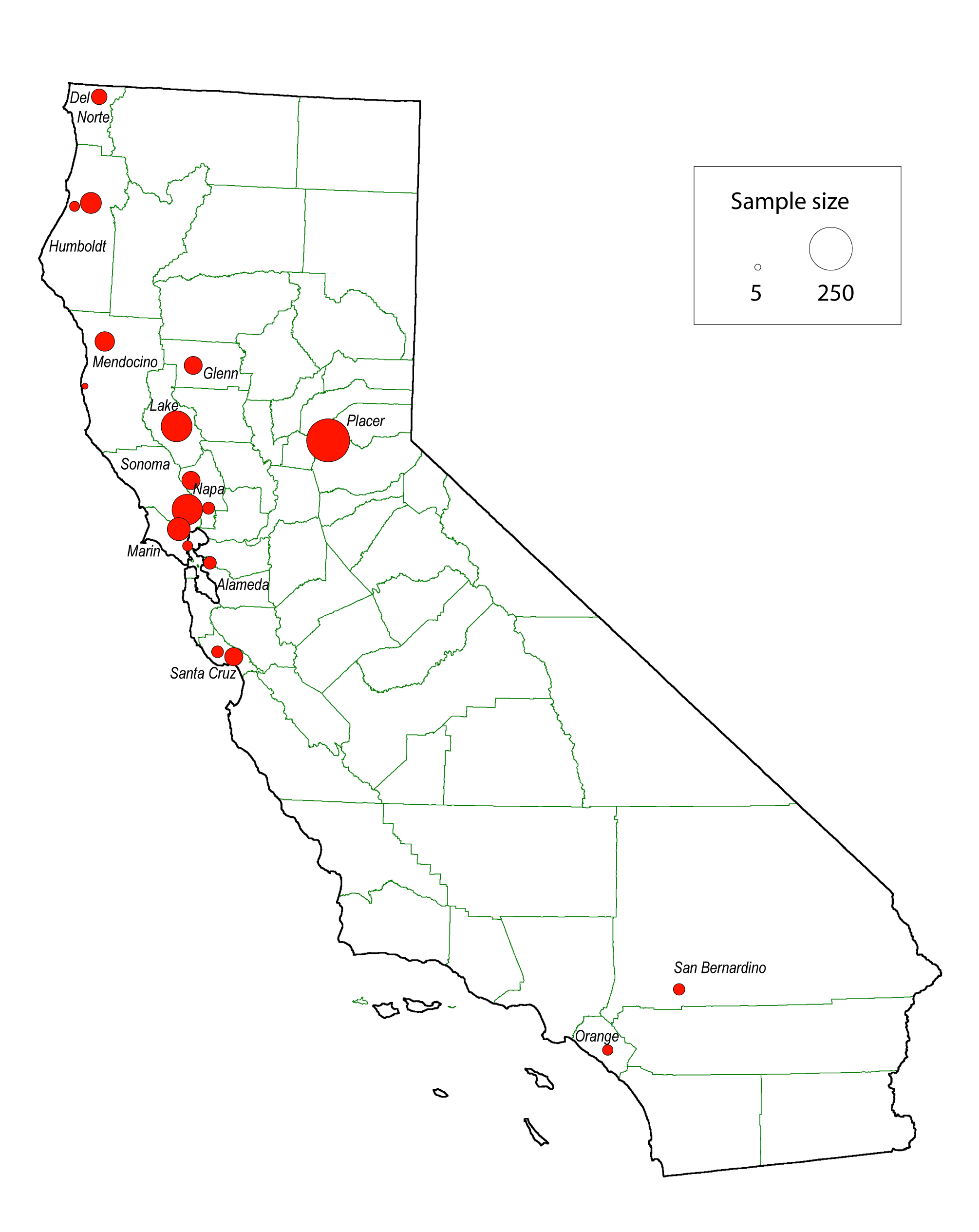

Supplement: S1 Fig — Dot size is proportional to the number of isolates collected. (TIF) [file pone.0135828.s009.tif]
